# Non-invasive joint decompression: An important factor in the regeneration of the bone marrow and disc recapture in temporomandibular arthropathies

**DOI:** 10.4317/medoral.22397

**Published:** 2018-09-28

**Authors:** Ivson-Souza Catunda, Belmiro-Cavalcanti-do Egito Vasconcelos, Marcus-Vinicius-Martins Corrêa, Marcelo-Freire Matos, Emerson-Filipe-de Carvalho Nogueira, Jorge-Alfonso Learreta

**Affiliations:** 1 DDS, MSc, Department of Oral and Maxillofacial Surgery, University of Pernambuco, Recife, PE, Brazil; 2 DDS, MSc, PhD, Department of Oral and Maxillofacial Surgery, University of Pernambuco, Recife, PE, Brazil; 3 DDS, Private clinical, Diamantina, MG, Brazil; 4 DDS, Private clinical, Salvador, BA, Brazil; 5 DDS, MSc, PhD, University of Buenos Aires, Buenos Aires, BA, Argentina

## Abstract

**Background:**

This article aims to demonstrate the importance of the TMJ (Temporomandibular Joint) decompression in the treatment of degenerative processes and disc displacements, reporting two clinical cases treated with orthopedic and decompressive correction of TMJ.

**Material and Methods:**

The studies reported in this article show patients with muscle and joint pain who were evaluated pre and post-treatment through MRI (Magnetic Resonance Irradiation) to follow-up bone marrow regeneration and TMJ disc placement. Transcutaneous electrical stimulation (TENS), measurement equipment and IO (Intraoral Orthotic) were used to evaluate and treat the patients. A critical review of literature has also been conducted to confront clinical outcomes.

**Results:**

Marrow bone regeneration and disc placement were observed in both patients.

**Conclusions:**

The use of measurement equipment associated with TENS to find the correct rest position of the Jaw an the use of IO to decompress the TMJ was an effective way to promote bone marrow regeneration and disc placement, consequently improving function and quality of life.

** Key words:**Descompression, surgical, orthotic devices, temporomandibular joint disorders, temporomandibular joint disc, occlusal splints.

## Introduction

**Figure 1:**
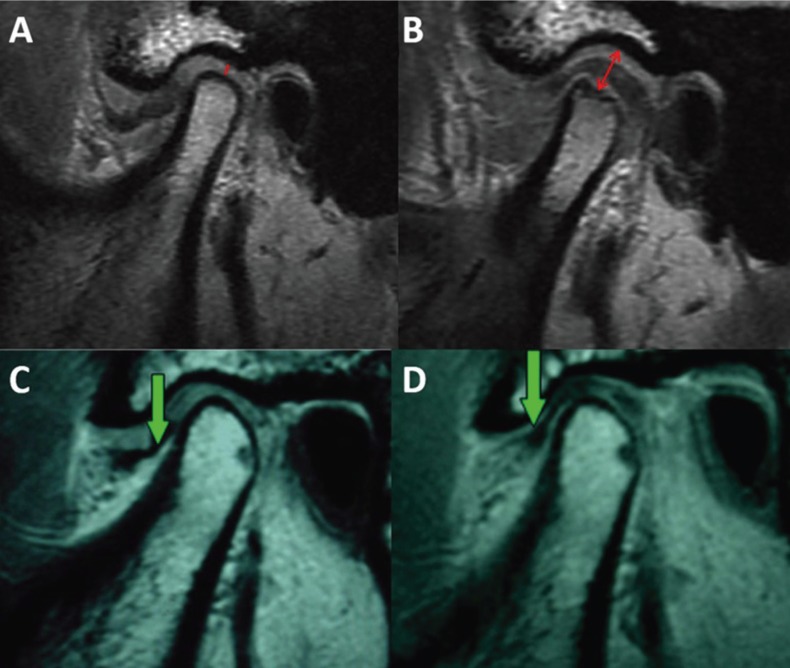
A and B) Magnetic Nuclear Resonance Imaging (MRI) taken from the article Hu *et al.*, (2017)(11)- demonstrating arthroscopic surgery with disc plication. A) Preoperative with decreased joint space and anteriorly displaced disc. B) Postoperative with repositioning of the disc and increased joint space with decompression (red line), where there will necessarily be occlusal alteration. It is also observed that the joint cartilage is reabsorbed by probable progression of arthropathy or disc compression on the cartilage. C and D) Images taken from Zhang *et al.*, (2010)(15]. C) MRI in the preoperative arthroscopic surgery evidencing anterior disc displacement (green arrow) and reduction of joint space. D) Postoperative of arthroscopic disc repositioning (green arrow) and increased joint space.

Changes in joint space may also be related to TMJ arthropathies, especially with disc displacements (1,2). Failure to keep the disc in its correct position is a common find in the literature when magnetic resonance imaging (MRI) is done in longer post-surgical follow-ups of disc plication, either by open or arthroscopic surgery. Few studies show good stability of the articular disc position, one example are the cases published by YANG (1-5), however, a decompressive orthopedic orthotic changing the condyle position is frequently observed, suggesting that the suture technique alone is not responsible for the stability of the disc repositioning but the decompression and increase of the joint space (Fig. 1).

This article aims to connect the importance of decompression of the temporomandibular joint in the treatment of degenerative processes and disc displacements, reporting two clinical cases treated with orthopedic and decompressive correction of TMJ.

## Case Report

Case 1

A 67-year-old woman with complaints of facial pain and tiredness in the masseter muscles for 7 years which got worse in the last year. She reported throbbing pains, previous TMJ clicks that evolved into crepitations. The pain got worse with chewing and during crises, it expanded to bilateral temporal region and nape. The patient was edentulous and used total dentures, removable upper and implant supported lower. She underwent numerous professional interventions such as myorelaxant splints, antidepressant medications, analgesics and anti-inflammatories without significant improvement.

**Figure 2:**
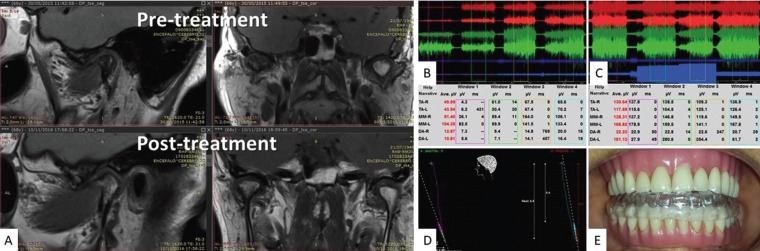
A) Upper images - Magnetic Nuclear Resonance (MRI) in PD (proton density) in slices (sagittal and coronal) in the pre-treatment, evidencing extensive bone marrow sclerosis in the right TMJ, facets, osteophyte and disc displacement with loss of the usual configuration. Lower images - MRI in PD (sagittal and coronal) in post-treatment control 18 months evidencing bone marrow area regeneration with hypersignal in the medullar of the right TMJ and increase of joint space by decompression. B) Joint decompression test before treatment (B) and post-treatment (C) with cotton rolls demonstrating maximum occlusion, right side roll occlusion, left side roll occlusion and bilaterally roll occlusion, In (B) with improvement muscle recruitment after increasing the vertical dimension. D) Take of the neurophysiological record from the mandibular three-dimensional / orthopedic positioning resulted from the neuromuscular deprogramming. E) view of occlusion with the use of the intraoral orthotic (IO).

Magnetic resonance imaging of the TMJ’s suggested bilaterally degenerative process, such as accentuated sclerosis of the right subchondral bone, osteophytes, displacement and sharpening of the articular disc and condylar repositioning. (Fig. 2A). The joint decompression test with electromyography suggested by Learreta (6) showed a need to recompose the lost vertical dimension even with the use of prostheses. The orthopedic neurophysiological position was obtained combined the MRI with the neuromuscular deprogramming with transcutaneous electrical stimulation (TENS). Once the rest position was established, a bite registration was performed taking in account the spatial position as seen in the MRI, as well as determination of neuromuscular rest position and free way space for orthotic construction (7,8). The patient made continuous use of the device, including for mastication, taking it out only for hygiene, for a total period of 16 months. A monthly evaluation protocol was established with a clinical, electromyography and kinesiography checkup, so that in neurophysiology could be addressed and the orthotic could be modified to keep orthopedic position up to date to the TMJ status (Fig. 2B-E). This protocol is due to the rehydration of the joint tissues during the decompression. Only two modifications of the device were needed in order to the pain disappear. There was also association in therapy with vitamin D3 supplementation, resveratrol, omega 3, N-acetylcysteine and low doses of naltrexone. All of them, used for six months.

Case 2

A 30-year-old woman complaining of joint pain and bilaterally clicking of both TMJ for 5 years, which got worse in the last two years. She also reported face pain when she woke up and left masseter tiredness. Chewing and speaking usually aggravated pain in the left TMJ and had louder clicks in the right TMJ. After numerous interventions such as functional orthopedics, myorelaxant splints, manual therapies, laser, acupuncture, antidepressants and various analgesics for about 2 years, the patient had not experienced any significant improvement of the condition. She also had a history of hypertension controlled with medications.

**Figure 3:**
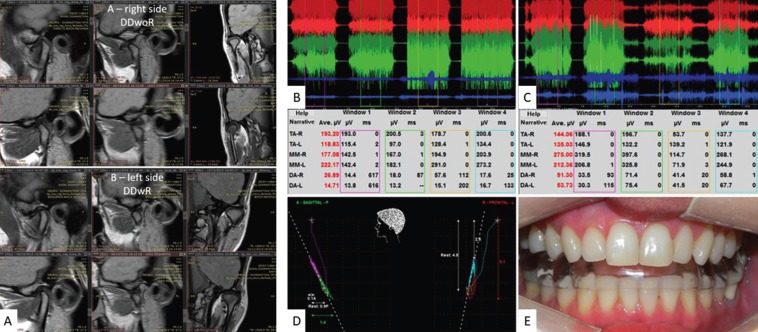
A) MRI in PD right side. Pretreatment (upper) in sagittal closed mouth with anterior disc displacement; open mouth without disc recapture and coronal with decreased joint space. Post-treatment (lower) in open and closed mouth with recapture of disc and increased joint space; coronal in the closed mouth showing joint decompression and repositioning of the articular disc. Left side MRI. Pretreatment (upper) in sagittal closed mouth with anterior disc displacement; open mouth with disc recapture and coronal with decreased joint space. Post-treatment (lower) in sagittal closed and open mouth with good disc placement and increased joint space; coronal showing joint decompression and repositioning of the articular disc in closed mouth. B) EMG/EGN. Measurement parameters before and during decompression treatment. Joint decompression test before treatment (B) and post-treatment (C) with cotton rolls demonstrating maximum occlusion, right side roll occlusion, left side roll occlusion and bilaterally roll occlusion, In (B) with improvement muscle recruitment after increasing the vertical dimension. D) Take of the neurophysiological record from the mandibular three-dimensional / orthopedic positioning resulted from the neuromuscular deprogramming. E) view of occlusion with the use of the intraoral orthotic (IO).

The same treatment protocol was used for joint decompression orthopedic neurophysiological alignment of the mandible. Follow up showed improvements in muscle recruitment based on Learreta´s EMG test (6,8). The orthotic was maintained for a period of 21 months of the treatment and beyond after discharge. The ending of the symptoms occurred after 5 months of treatmen and there was a significant improvement after 60 days of decompression. Four different devices were progressively used because there was a greater need for articular space. Later after clinical discharge, the patient went to the second phase of treatment with three-dimensional volumetric orthodontics in order to replace the orthotic with teeth, keeping the vertical dimension and, therefore, joint spaces (Fig. 3).

## Discussion

Transient improvements in joint symptomatology of temporomandibular disorders with the use of splint / stabilizing plaques have been well known, but little is known about the real reason for efficacy in terms of resolution of symptoms. Instead, symptomatic improvement is observed for some two to three months and then, gradual return of symptoms.

It has been shown that the key treatment for the pains that involve the various joints of the body, including TMJ, is to reduce the overload and allow joint movement as soon as possible (1-3,9,10). Dystrophic calcification / medullar sclerosis seen on magnetic resonance imaging, when present in TMJ indicates an advanced stage of arthropathy (11,12), but sometimes representing little pain or signs typical of temporomandibular disorders (13), probably due to increased joint space and decompression caused by the alteration of the anatomical form of the condyle, fossa and joint eminence.Regarding the displacement of the TMJ joint disc, it has been suggested that the disc repositioning is conditioned by the increase of the joint space with mandibular orthopedic modification called joint decompression (2,3), and the increase of space is the main responsible for joint disc maintenance. Thus, the long-term stability of the disc tissues is conditioned to the increase of the joint space, consequently the rehydration and increase of volume of the tissues, as well as the need to occupy the increased space between the jaws connected to the resting caused by the deprogramming, that’s why the IO / interocclusal orthotic is used.

Necessarily the neurophysiological response generated by the occlusal orthotic need to be evaluated. For this purpose, electromyography readings were performed as proposed by Learreta (6) and determined the need to recover the vertical dimension. The anteroposterior mandible positioning was also measured by the axiality of the mandibular movements and masticatory cycles through electrognatography / kinesiography. We believe that joint decompression is a primordial factor, that is part of the necessary tools to control the processes involved in degenerative diseases and even in disc displacements, such as reactive arthritis and autoimmunity. In the first report, a protocol was established to improve immunity with vitamin D3, resveratrol, omega 3 and low doses of naltrexone for a minimum period of 6 months. However, in some traumatic cases only the orthopedic and decompressive correction of the TMJ may be needed.

Both cases started with neuromuscular deprogramming with orthotic devices, but the patient with medullar sclerosis and reactive arthritis was treated with antibio-tic, low dose nauxetron to improve immunity (Younger) and supplementation.

All the interventions in this proposed model follow the recommendations of the literature regarding the less invasive and reversible treatment. It is up to the dentist who conducts the case, planning in a second phase after orthopedic correction of the mandible, reprograming the dental occlusion according to the established vertical dimension, without modifying the condylar positioning, so that it will be possible to perform the three-dimensional volumetric orthodontics (10), prosthetic rehabilitations or even orthognathic surgery. After termination of the first phase, the patients begin the secound phase with maintenance of the vertical dimension given by the device. The dentate patient was sent to perform volumetric orthodontics, while the endentulous patient refitted the prostheses based on the established height.

The decompression of the temporomandibular joint through the deprogramming of the musculature and neurophysiological orthopedic correction of the mandible presents great potential in the treatment of the degenerative processes and disc displacement of the TMJ. Clinical research controlled and evaluated by magnetic resonance imaging should be performed to confirm these findings.
